# IgE re-programs alternatively-activated human macrophages towards pro-inflammatory anti-tumoural states

**DOI:** 10.1016/j.ebiom.2019.03.080

**Published:** 2019-04-05

**Authors:** Giulia Pellizzari, Coran Hoskin, Silvia Crescioli, Silvia Mele, Jelena Gotovina, Giulia Chiaruttini, Rodolfo Bianchini, Kristina Ilieva, Heather J. Bax, Sophie Papa, Katie E. Lacy, Erika Jensen-Jarolim, Sophia Tsoka, Debra H. Josephs, James F. Spicer, Sophia N. Karagiannis

**Affiliations:** a St. John's Institute of Dermatology, School of Basic and Medical Biosciences, King's College London, Guy's Hospital, London SE1 9RT, United Kingdom; b Department of Informatics, Faculty of Natural and Mathematical Sciences, King's College London, Bush House, London WC2B 4BG, United Kingdom; c Institute of Pathophysiology and Allergy Research, Center for Pathophysiology, Infectiology and Immunology, Medical University Vienna, Austria; d Department of Comparative Medicine, The Interuniversity Messerli Research Institute of the University of Veterinary Medicine Vienna, Medical University Vienna and University Vienna, Vienna, Austria; e School of Cancer & Pharmaceutical Sciences, King's College London, Bermondsey Wing, Guy's Hospital, London SE1 9RT, United Kingdom; f Breast Cancer Now Research Unit, School of Cancer & Pharmaceutical Sciences, King's College London, Guy's Cancer Centre, London, United Kingdom; g Guy's and St Thomas' NHS Trust, Department of Medical Oncology, London, United Kingdom

**Keywords:** Cancer immunotherapy, IgE, Macrophages, Alternatively-activated macrophages, AllergoOncology, Pellizzari et al.

## Abstract

**Background:**

Antibody Fc-driven engagement of macrophages is critical for evoking cellular activation and effector functions and influencing tumour-associated macrophage (TAM) recruitment. We previously reported that IgE class antibodies promote restriction of cancer growth in rodent models associated with significant TAM infiltration. However, the human macrophage-associated IgE-Fc Receptor (FcεR) axis remains unexplored. We investigated the effects of anti-tumour IgE stimulation on human macrophage activation.

**Methods:**

Human blood monocyte-differentiated quiescent (M0), classically-(M1) and alternatively-(M2) activated macrophages were crosslinked with IgE and polyclonal antibodies to mimic immune complex formation. We examined surface marker expression, cytokine secretion, protein kinase phosphorylation and gene expression in IgE-stimulated macrophages and IgE antibody-dependent macrophage-mediated cytotoxicity (ADCC) against tumour cells.

**Findings:**

A proportion (40%) of M2 and (<20%) M0 and M1 macrophages expressed the high-affinity IgE receptor FcεRI. IgE crosslinking triggered upregulation of co-stimulatory CD80, increased TNFα, IFNγ, IL-1β, IL-12, IL-10, IL-13, CXCL9, CXCL11 and RANTES secretion by M0 and M2 and additionally enhanced MCP-1 by M2 macrophages. IgE-stimulated M1 macrophages retained secretion of pro-inflammatory cytokines. IgE crosslinking enhanced the FcεRI-dependent signalling pathway, including phosphorylation of the Lyn kinase, ERK1/2 and p38 in M2 macrophages and upregulated *Lyn* gene expression by M1 and M2 macrophages. Anti-tumour IgE engendered ADCC of cancer cells by all macrophage subsets.

**Interpretation:**

IgE can engage and re-educate alternatively-activated macrophages towards pro-inflammatory phenotypes and prime all subsets to mediate anti-tumour functions. This points to IgE-mediated cascades with potential to activate immune stroma and may be significant in the clinical development of strategies targeting tumour-resident macrophages.

**Research in context**

**Evidence before this study**

Tumour-associated macrophages (TAMs) with alternatively-activated pro-inflammatory features represent a significant component of the immune infiltrate in the tumour microenvironment (TME). Monoclonal antibodies may influence macrophage activatory states and functions, however the impact of antibody isotype on engagement of different macrophage subsets remains unclear. We have previously reported that IgE class antibodies recognising tumour-associated antigens elicit anti-tumour immune responses in rodent models. IgE-mediated anti-tumour effects were associated with an increased influx of macrophages into tumour lesions likely engendered through IgE Fc-mediated cascades.

**Added value of this study**

In this study we demonstrate that human IgE is responsible for potentiating Fc-mediated activation of alternatively-activated macrophages that leads to enhanced FcεRI-dependent signalling and a cytokine signature that bears characteristics normally associated with classically-activated, pro-inflammatory macrophages. Moreover, we show for the first time that IgE can prime quiescent, classically-activated and alternatively-activated macrophages to mediate anti-tumour cell cytotoxic effects.

**Implications of all the available evidence**

This cross-talk of IgE class antibodies with human macrophages may be significant for the clinical development of cancer immunotherapies that exploit the plasticity of these often tissue- and tumour-resident cells to restore their antitumor properties. Our findings thus point to IgE class antibodies as mediators of a cascade with potential to activate and re-educate immune stroma against cancer.

Alt-text: Unlabelled Box

## 1 Introduction

Tumour-associated macrophages (TAMs) form a significant proportion of the immune cell infiltrate and are thought to play important roles in defining the pro- or anti-tumour status of the tumour microenvironment (TME). The mechanisms by which macrophages can influence tumorigenesis, though, has been controversial: these cells represent a multi-faceted immune compartment and their contributions in shaping a pro- or anti-tumour environment heavily depend on their polarisation status [1]. While the conventional binary model of macrophage polarisation of classically-activated (M1) and alternatively-activated M2 subsets may be imprecise, generally tumour-associated macrophage infiltrates are thought to favour an alternatively-activated M2-like phenotype. TAMs, featuring M2 characteristics, have been demonstrated to permit or promote tumour growth and their presence in the tumour tissue has been associated with poor prognosis in some solid tumours of various origins, such as melanoma, head and neck, lung, colorectal and breast cancers [2]. For these reasons, TAMs are now considered a potential target for cancer immunotherapy [[3], [4], [5], [6]].

Several anti-cancer approaches targeting macrophages have been explored aiming to either inhibit their tumour-supporting functions or to promote their anti-tumour effects. More specifically, macrophage-centred anti-cancer strategies can include macrophage depletion, modification of macrophage recruitment and macrophage re-polarisation or re-programming. Macrophage reprogramming involves the suppression of alternatively-activated (M2) macrophages or, possibly, the stimulation of an M1-biased phenotype in the TME [7]. The development of a monoclonal antibody targeting the pattern recognition scavenger MARCO [8] is the first antibody therapeutic approach designed to re-programme TAMs.

The demonstration that re-polarisation of macrophages can be achieved through monoclonal antibody treatment opened the door to the development of new therapeutics targeting macrophage surface proteins (specifically those involved in maintaining an M2-like phenotype). We previously demonstrated that treatment with MOv18 I.E. a monoclonal human/chimeric antibody engineered with Fc regions of the IgE class and specific for a tumour-associated antigen (TAA) (Folate Receptor alpha, FRα), triggered an immune-activatory TNFα/MCP-1 axis in tumours, which resulted in recruitment of macrophages into tumour lesions and was associated with significantly-reduced tumour growth [9], in a syngeneic immunocompetent lung metastases rat model. These findings suggested that IgE antibodies may activate and recruit macrophages towards tumours and thus may represent a treatment approach capable of engaging these cells against cancer [3,10]. However, while activation of mast cells and subsequent release of pro-inflammatory mediators have been largely described [11,12], very little is known regarding IgE-Fcε Receptor interactions and their downstream effects on macrophage activation and functions, especially with regards to distinct macrophage subpopulations [13].

In our study we investigated how a monoclonal antibody (SF-25 I.E. targeting a TAA, can engage and activate human macrophages of different activation states. We investigated human macrophage expression of IgE receptors, the potential effects of IgE crosslinking on surface marker expression, soluble mediator release, phosphorylation and gene expression of kinases known to be associated with FcεR signalling. Furthermore, we investigated whether macrophages of different polarisation states can trigger effector functions against cancer cells by anti-tumour IgE, with the aim of ascertaining the potential of antibodies of the IgE class to promote macrophage-mediated anti-tumour activity.

## 2 Materials and methods

### 2.1 Isolation of human peripheral blood mononuclear cells (PBMC)

K_2_EDTA-spray coated collection tubes were used to collect peripheral venous blood (50 ml) from healthy donors. To isolate PBMCs, equal volumes of blood and 2% FCS/2 mM EDTA were gently mixed to a final volume of 30 ml and gently pipetted on top of 15 ml of Ficoll-Paque™ PLUS density gradient in a 50 ml conical tube. The tube was then centrifuged at 1200 x g with slow acceleration and no brake at room temperature (RT) for 20 min. The plasma interface was collected and washed with PBS at 600 x g at 4 °C for 10 min. The erythrocytes present in the sample were lysed with RBC lysis buffer for 5 min at RT, followed by washing with PBS + 2% FCS/2 mM EDTA. Human samples were collected with informed written consent, in accordance with the Helsinki Declaration. Study design was approved by the Guy's Research Ethics Committee, Guy's and St. Thomas' NHS Foundation Trust. Peripheral blood was also obtained through the UK National Health System (NHS) Blood and Transplant system from anonymous donor leukocyte cones.

### 2.2 Isolation of human monocytes from peripheral blood

Isolated PBMCs were passed through a 40 μm cell strainer to remove cell clumps. Next, PBMCs were transferred in a new vial and washed with PBS at 600 x g for 5 min. After discarding the supernatant, cells were resuspended in 400 μl of MACS buffer (PBS + 0.5% FCS/2 mM EDTA). Human monocytes were isolate by negative selection, following the Pan Monocyte Isolation Kit protocol (Miltenyi Biotec). The purity of monocytes was evaluated by flow cytometry staining with a BV786-conjugated monoclonal antibody anti-CD14 and BV711-conjugated anti-CD16. Acquisitions were performed using a BD FACS Canto™ II at the Biomedical Research Centre Flow Cytometry Core, King's College London.

### 2.3 Differentiation of human macrophages *ex vivo* from peripheral blood monocytes

Isolated monocytes were resuspended in RPMI 1640 2% FBS to promote adhesion and cells were seeded in 6-well plates at a density of 1–1.5 × 10 [6] cells/ml; each well was loaded with 2 ml of cell suspension and plates were placed in a tissue culture incubator. After 2 h of incubation, medium was carefully removed. Each well was washed with 1 ml of warm sterile PBS and replenished with 2 ml of RPMI 1640 supplemented with 10% FCS and penicillin/streptomycin with the addition of 20 ng/ml of M-CSF (Monocyte Colony Stimulating Factor) to allow monocyte to grow *ex vivo*. Thereafter, every 3 days, half of the volume in each well fresh were replaced with media containing 25 ng/ml GM-CSF (for M1) or 40 ng/ml M-CSF (for M2). On Day 7, 20 ng/ml of Interferon-Gamma (IFNγ) and 100 ng/ml of Lipopolysaccharide (LPS) were added to GM-CSF cells for 72 h. M-CSF cells were instead added with 20 ng/ml of Interleukin-4 (IL-4) for 72 h.

### 2.4 Flow cytometric assessment of antibody binding to antigen and cell surface receptors

Binding of SF-25 IgE and IgG1 to FcεR- and FcγR-expressing cells or antigen-expressing cells was evaluated by using 10^5^ cells per sample. Cells were detached with PBS + 5 mM EDTA, resuspended in PBS + 2% FBS (FACS buffer) and incubated with 5 μg/ml of primary antibody for 30 min at 4 °C, followed by a wash with 3 ml of FACS buffer (spinning at 500 g for 5 min). Cells were incubated with the secondary antibody goat anti-human IgE–fluorescein isothiocyanate (FI-3040; Vector Laboratories) or goat anti-human IgG1–fluorescein isothiocyanate (FI-3080; Vector Laboratories) at 30 μg/ml in FACS buffer for 30 min at 4 °C, followed by one wash as above. Samples were resuspended in 100 μl of FACS buffer and analysed with a BD FACS Canto™ II.

### 2.5 Detection on endogenous IgE on differentiated macrophages

M0, M1 and M2 monocyte-derived (MDM) macrophages were detached with Accutase and 1 × 10^5^ cells were aliquoted in FACS tubes in RPMI1640 medium supplemented with 2% FBS. Cells were incubated with 5 μg/ml of IgE antibody for 30 min at 4 °C. Macrophages were then washed with PBS to remove any unbound antibody. Cells were then treated with 1 μg/ml of goat polyclonal anti-human IgE–fluorescein isothiocyanate (FI-3040; Vector Laboratories) for 30 additional mins at 4 °C. Cells were washed again with PBS by centrifugation for 5 mins at 400 ×*g* and resuspended in FACS buffer, before analysing them on a BD FACS Canto™ II.

### 2.6 IgE crosslinking on human macrophages

M0, M1 and M2 (MDM) macrophages were incubated with 5 μg/ml of IgE antibody for 30 min at 37 °C by adding the antibody solution directly onto the cell culture plate. Cells wells were washed with PBS to remove any unbound antibody. Cells were then treated with 1 μg/ml of goat polyclonal anti-human IgE–fluorescein isothiocyanate (FI-3040; Vector Laboratories) for 30 more mins at 37 °C. Cells were harvested 4 h after stimulation to examine soluble mediator release and 24 h after stimulation to investigate surface marker expression.

### 2.7 Magnetic luminex assay of macrophage supernatants for cytokine detection

Macrophage supernatants were collected 4 h post-stimulation, centrifuged at 600 x g to remove floating cells and diluted 1:1 with sterile PBS. The multiplex assay was performed with the Magnetic Luminex Kit following the protocol instructions. The plate was sealed and left on the shaker up to 90 min until ready to be analysed on a Luminex™ analyzer.

### 2.8 Flow cytometry assessment of surface protein expression on macrophages

The day after the treatments, cells were detached with Accutase (Sigma). Plates were incubated for 10 min at 37 °C to facilitate detachment. After the incubation, 1 ml of RPMI 1640 supplemented with 10% FCS and penicillin/streptomycin was added to each well. Cells were counted, washed with PBS by centrifugation at 400 x g for 5 min and resuspended at a concentration of 1 × 10^6^ cells/ml. 5 ml poly-propylene tubes were used for staining and flow cytometry acquisition. To each sample, 1 μl of each of the staining antibody solution was added, and cells were incubated for staining at 4 °C for 40 min, before being washed with 2 ml of FACS buffer and centrifuged as above. Cells were resuspended in 100 μl of PBS only and stained for live/dead cell discrimination with LIVE/DEAD**®** Fixable Dead Cell Stain Near-IR and incubated at room temperature for 30 min in the dark. Tubes were added with 2 ml of PBS and washed by centrifugation as above, before being resuspended in 200 μl of FACS buffer and acquired at BD FACS Canto™ II.

### 2.9 Investigation of gene expression *via* real time quantitative PCR (RT-qPCR)

cDNA samples were diluted 1:4 with RNAse/DNAse free water and 2 μl were distributed in triplicate on a fast-optical 96-well reaction plate. A qPCR master mix was prepared by mixing 10 μl of TaqMan gene expression master mix, 1 μl of GAPDH Probe, 1 μl of the GOI Probe (T cell (or transmembrane) immunoglobulin and mucin domain protein 3 *Tim-3* or *Lyn* kinase) and 6 μl RNAse free water. The qPCR master mix was added to every well containing DNA. Negative qPCR controls included cDNA-free wells. 40 cycles of RT-qPCR were performed.

### 2.10 Cytotoxicity/phagocytosis (ADCC/ADCP) assay to study IgE antibody functions in activating macrophages against cancer cells

ADCC/ADCP assays were performed using a protocol adjusted from a previously-described method [14]. Briefly, target tumour cells were labelled with Carboxyfluorescein succinimidyl ester (CFSE) the day before performing the assay. For this, cells were detached with trypsin and washed once in RPMI 1640 complete medium. Cells were then resuspended in 100 μl PBS, and 0.75 μl of 0.5 mM CSFE was added for every 1 × 10^6^ cells. Cells were incubated at 37 °C for 10 min and the reaction was stopped with one wash in ice-cold RPMI 1640 standard medium. Normal culture conditions were resumed overnight. The following day, CFSE-labelled tumour cells were detached using PBS + 5 mM EDTA, washed in RPMI 1640 standard medium and re-suspended to 1 × 10^5^ cells/100 μl culture medium. Macrophages were detached with Accutase (Sigma), re-suspended in culture medium and mixed with the tumour cells at a 10:1 ratio. Cells were placed in FACS tubes and co-incubated at 37 °C for 3 h in the presence of either PBS or 5 μg/ml of either SF-25 IgE or control antibody. After incubation, cells were washed in FACS buffer and macrophages were identified by staining mixed cell populations with 5 μg/ml of anti-human CD206-APC/Cy7-conjugated antibody to detect M1 or anti-human CD163-PE/Cy7-conjugated for M2 for 30 min at 4 °C. Cells were washed once and re-suspended in 200 μl FACS buffer for immediate acquisition using a dual laser FACSCanto™ (Becton Dickinson). Every sample was treated with 100 μl of DAPI (100 ng/ml), mixed using a vortex and acquired immediately. Tumour cell killing was calculated on the percentage of DAPI^+^ tumour cells and the total number of tumour cells in SF-25 antibody-treated samples compared to negative controls. Analysis of flow cytometry data was performed by FlowJo (TreeStar Inc) software.

### 2.11 Analysis of the phosphorylation profile of kinases

Proteome Profiler Human Phospho-Kinase Array (R&D Systems) were incubated with 400 μg lysate overnight at 4 °C. The following day, chemiluminescent detection was performed according to manufacturer's indications. Densitometry analysis was conducted using ImageJ.

### 2.12 Study of FcεRI pathway in macrophages

Pathway analysis was performed using the Reactome Pathway Analysis tool [15].The set of genes of phosphorylated kinases used for input was the combined superset from those perturbed under both IgE stimulation of M1 and M2. In order to determine the ability of each gene product to discriminate between the observed differences in M1 and M2 phosphorylation, the difference in fold change between M1 and M2 was calculated. The M1/M2 difference in fold change was used to weight each gene when inferring implicated pathways. Kinases were considered “perturbed” by IgE-crosslinking when the *t*-test *p*-value performed on the means of each condition (baseline/IgE crosslinked), resulted in a *P* value of <0.05. A False Discovery Rate of <0.05 and a minimum gene set size of 4 was used to filter the results. Overly general pathways and processes were manually removed. Version 67 of the Reactome database was used. The KEGG database was used to depict the structure of the FcεRI pathway.

### 2.13 Statistical methods and analyses of publicly-available databases

All statistical analyses were performed using GraphPad™ Prism Software (version 6.0). Error bars represent SD and SEM in *in vitro* and in *ex vivo* evaluations. Clinical associations of gene expression were assessed using publicly-available data in Kaplan-Meier Plotter (http:/kmplot.com/analysis/). One way-ANOVA was performed in combination with Bonferroni's or Tukey's post-test to determine statistical significance of the data.

## 3 Results

### 3.1 Human monocyte-derived macrophages express typical M1 and M2 differentiation surface markers and express IgE Fc receptors

**Fig. 1:**
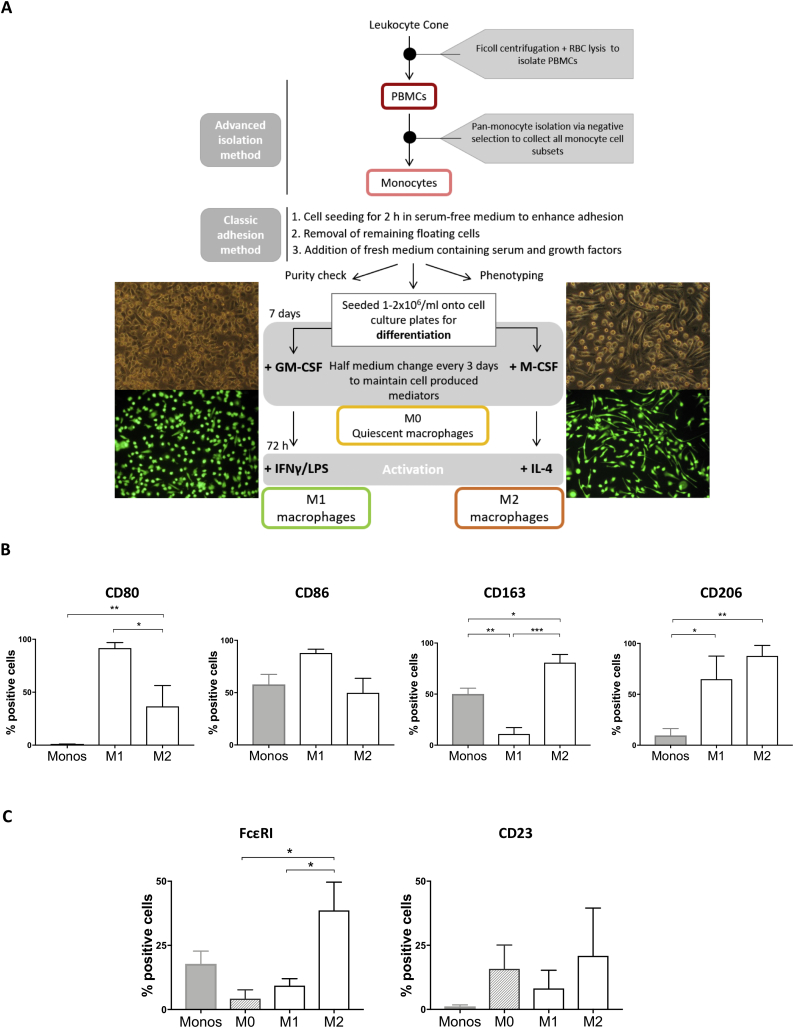
M1 and M2 macrophages are obtained through *ex vivo* differentiation of human monocytes. (a) Flowchart depicting the isolation and cell culture methodology to derive M1 and M2 macrophages *ex vivo*. (b) Flow cytometric analysis of M1 and M2-typical surface markers CD80, CD86, CD163, CD206 representing the percentage of positive cells to anti-human CD80-BV395, anti-human CD86-BV737, anti-human CD163-BV605, anti-human CD206-PE/Cy7 antibody respectively (average of 4 independent experiments ± SEM). (c) Detection of FcεRI-II *via* flow cytometry on monocytes, M0, M1 and M2 *ex vivo* differentiated macrophages. Data represent the percentage of positive cells to anti-human FcεRI-PE, anti-human CD23-BV421 antibodies (average of 4 independent experiments ± SEM). Statistical analysis obtained performing One-way ANOVA coupled with Tukey's post-test (*** *p*-value<.0005, ** p-value<.005, * p-value<.05).

**Supplementary Fig. 1:**
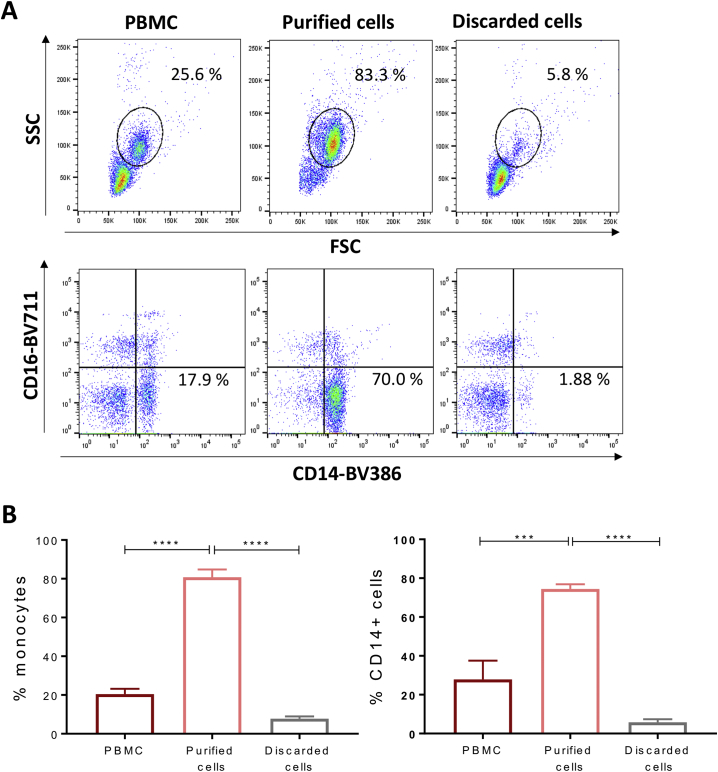
Gating strategy for monocyte purity evaluation (a) Representative flow cytometry plots depict cell populations in sample fractions. (b) Level of monocyte purity in the isolated samples (*n* = 4) *via* CD14 and CD16 staining. Values are represented as percentage of positive cells (average of *n* = 3 independent experiments ± SEM).

To evaluate the impact of Fc engagement of IgE on human macrophage populations, we firstly confirmed the characteristics of classically-activated (M1) and alternatively-activated (M2) subsets [2]. Human primary monocytes isolated from peripheral blood by negative selection and magnetic bead purification [16,17] comprised the three classical (CD14^++^, CD16^−^), intermediate (CD14^+^, CD16^+^) and alternative (CD14^−^, CD16^++^) subsets (Supplementary Fig. 1A). Non-adherent, non-monocytic cells were removed after two hours of seeding in serum-free medium, consistently yielding >80% monocyte purity based on CD14 and CD16 expression and physical parameters (Supplementary Fig. 1A-B). Macrophages were differentiated over 7 days (Fig. 1A), followed by cytokine stimulation (IFNγ+LPS for M1, IL-4 for M2) for 72 h, to obtain classically (M1) and alternatively (M2) activated macrophage subsets, respectively. Consistent with previous reports [18], differentiated populations showed the expected morphology profiles: fried egg-shape like GM-CSF and M1 macrophages, and fibroblast-like, needle-shaped M-CSF macrophages, which further elongated after treatment with IL-4 (Fig. 1A).

We ascertained expression of key markers of macrophage differentiation (co-stimulatory molecules CD80, CD86 for M1; the scavenger receptor cysteine-rich family member CD163 (or M130), and the mannose receptor CD206 for M2) (Fig. 1B). Consistent with previous studies, peripheral blood monocytes expressed CD86 but not CD80 [19]. A high proportion of M1 macrophages expressed both CD80 and CD86. We found expression of CD163 [20] on 50% of freshly isolated monocytes and on a high proportion of alternatively-activated (M2) macrophages. Furthermore, 10% of primary monocytes and the majority of M1 and M2 macrophages expressed CD206, in line with previous reports [21]. These findings confirmed the differentiation of classically- and alternatively-activated macrophage subsets from human monocytes.

Very little information is available regarding IgE receptor expression for distinct macrophage subpopulations [13]. We found that 20% of peripheral blood monocytes expressed the high affinity IgE Fc receptor FcεRI, while 1–2% expressed the low affinity receptor FcεRII (CD23), in line with previously published studies [22,23] (Fig. 1C). M2 macrophages displayed the highest levels of cell surface expression of FcεRI (40% of cells, *n* = 4), while CD23 expression was expressed by 15–20% of all macrophage phenotypes (M0, M1, M2).

**Supplementary Fig. 2:**
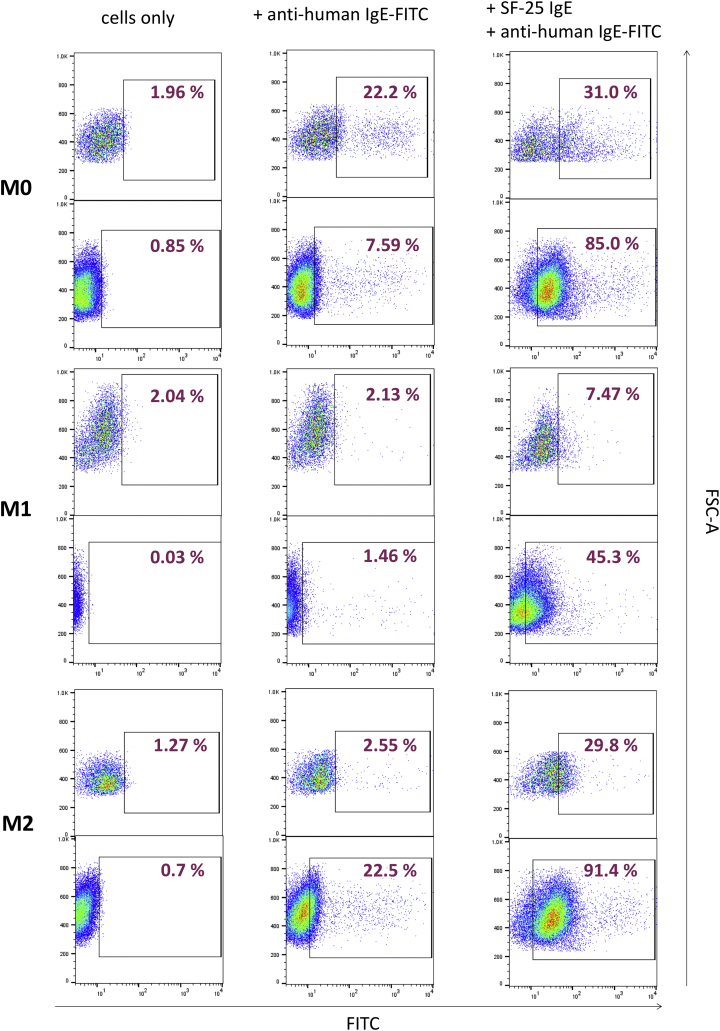
Surface levels of endogenous IgE on differentiated macrophages Representative flow cytometry plots of two independent experiments for quantification of endogenous IgE bound to differentiated macrophages, treated with anti-human IgE-FITC only or SF-25 IgE and anti-human IgE-FITC combined. FITC-positive cells are gated and relative percentage to whole cell sample is depicted.

Based on the high affinity of IgE for cognate Fc receptors and its slow dissociation rates most notably following binding to FcεRI on immune cells, we hypothesised that some endogenous IgE may be retained by effector cells *ex vivo* in the absence of antigen engagement and immune complex formation [24,25]. An anti-human IgE FITC-conjugated antibody was used to detect endogenous IgE and also following treatment with anti-tumour IgE (SF-25) to understand whether free IgE Fcε receptors were available for exogenous IgE to bind and, to crosslink these on the surface of macrophages [[26], [27], [28]]. Over 20% of M0 macrophages were detected to still have cell surface-bound endogenous IgE when incubated with anti-IgE antibody alone (Supplementary Fig. 2). Both M1 and M2 macrophages showed detectable but lower endogenous IgEs on their surface. All cell subsets could also bind exogenous IgEs.

Together, as previously demonstrated with primary human monocytes [29,30], these findings indicate that IgE Fc receptors were not saturated on the surface of human macrophages and that exogenous IgEs could still engage with these cognate Fcε receptors despite the presence of endogenously-bound IgE.

### 3.2 IgE crosslinking on macrophages upregulates M1 surface marker CD80 levels and triggers secretion of a distinct cytokine chemokine signature by M0 and M2 macrophages

We previously reported that systemic administration of rat anti-tumour IgE was associated with recruitment of CD80+ macrophages into syngeneic tumours of immunocompetent rats [9]. Here we investigated whether IgE stimulation might impact on human classically and alternatively activated macrophage surface marker expression. Human macrophage subsets were incubated with either anti-tumour (SF-25) IgE or the hapten-specific antibody NIP IgE. IgE-Fcε receptor crosslinking was triggered by polyclonal anti-human IgE. Crosslinking of IgE on macrophages did not affect CD14, FcεRI or CD23 surface expression, while FcγRIII (CD16) expression by M2 macrophages was reduced with crosslinking of both SF-25 and NIP IgE (Supplementary Fig. 2). Surface expression of CD80 was upregulated on M0 and M2 macrophages when cell bound IgE was crosslinked but remained unaltered on M1 macrophages. Neither IgE engagement nor crosslinking affected CD163 or CD206 surface expression on any of the cell subsets.

**Fig. 2:**
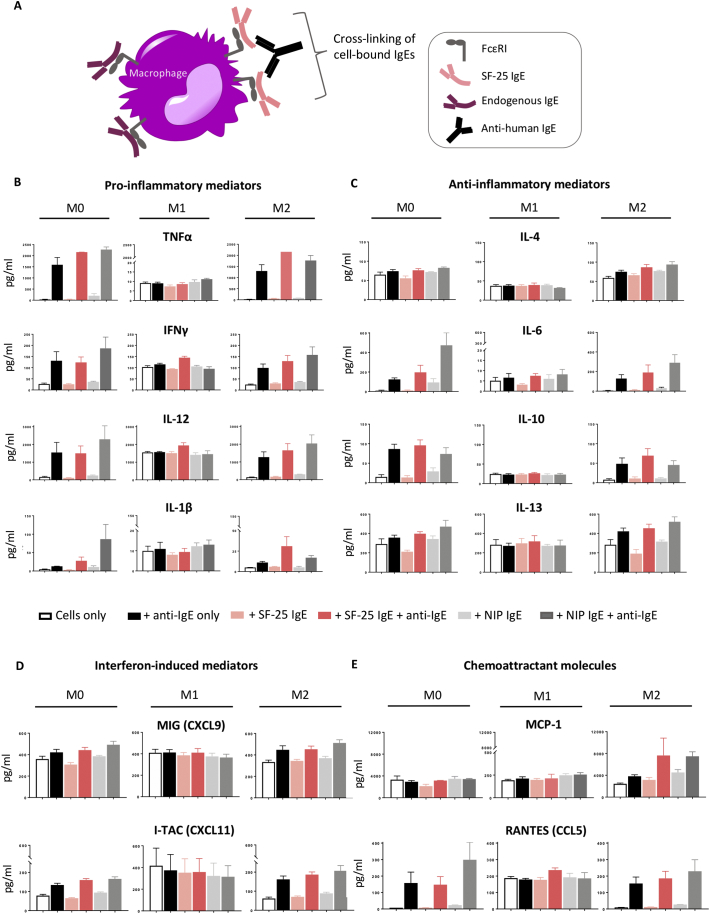
Cross-linking of cell-bound IgE regulates macrophage secretion of soluble mediators. (a) Schematic representation of the cross-linking of IgE antibodies on macrophages. (b-e) Differentiated macrophages were incubated with SF-25 IgE or NIP IgE antibody alone for 30 min at 37 °C or followed by a second 30 min incubation with anti-human IgE antibody in order to cross-link the cell-bound IgEs. (b) Quantification of Pro-inflammatory cytokines TNFα, IFNγ, IL-12, IL-1β, (c) Anti-inflammatory cytokines IL-4, IL-6, IL-10, IL-13, (d) Interferon Gamma-induced chemokines MIG (CXCL9) and I-TAC (CXCL11) and (e) Macrophage chemoattractant chemokines MCP-1 (CCL2) and RANTES (CCL5) levels were analysed *via* Luminex magnetic assay. Values are expressed as pg/mL (average of two independent experiments ± SEM).

Based on previous evidence that IgE can influence macrophage recruitment and activation in rodent models [9], we investigated whether IgE crosslinking of Fcε receptors on the surface of different human macrophage subsets could influence soluble mediator release (Fig. 2A):

Pro-inflammatory cytokines TNFα, IFNγ, IL-12, IL-1β: Crosslinking of IgE led to a drastic increase (from 100 to approximately 2000 pg/ml) in TNFα secretion in both M0 and M2 macrophages, while IgE did not affect the low TNFα levels produced by M1 cells (Fig. 2B). IgE crosslinking enhanced IFNγ secretion by both M0 and M2 subsets. M1 macrophages showed a 4-fold higher baseline levels of IFNγ compared to the other subsets and these remained stable upon activation *via* IgE. Furthermore, M0 and M2 macrophages increased IL-12 production from 100 to approximately 1500–2000 pg/ml when crosslinked with IgE/anti-IgE. M1 cells featured a greater baseline level of IL-12 (*ca.* 1500 pg/ml), which remained unaffected upon IgE treatment. Baseline concentration of IL-1β were very low in all three cell subsets. Crosslinking of cell-bound IgE triggered increased IL-1β secretion in M0 and M2 macrophages, while stimulation did not influence production by M1 macrophages.

Anti-inflammatory cytokines IL-4, IL-6, IL-10, IL-13: IL-4 production slightly increased upon IgE crosslinking in both M0 and M2 subsets and remained unaffected in M1 cells. Baseline levels of IL-10 and IL-13 were similar between macrophage subsets, however IgE crosslinking upregulated IL-10 and IL-13 release by M0 and M2 subsets but not by M1 macrophages. IL-6 secretion remained unchanged in M1 macrophages but increased upon treatment with IgE only and with IgE/anti-IgE crosslinking in both M0 and M2 subsets (Fig. 2C).

Interferon Gamma-induced chemokines MIG (CXCL9) and I-TAC (CXCL11): In M1 macrophages, secretion of MIG (CXCL9) and I-TAC (CXCL11) remained unchanged (*ca.* 400 pg/ml) with IgE stimulation, while crosslinking of IgE in M0 and M2 macrophages led to modest increases. Levels of I-TAC (CXCL11) were 4-fold greater in M1 macrophages (*ca.* 400 pg/ml) compared to M0 and M2 at baseline, however, IgE crosslinking increased secretion of I-TAC (CXCL11) by M0 and M2 cultures from *ca.* 80 to *ca.* 160 pg/ml in M0 cells and from 50 to *ca.* 200 pg/ml in M2 macrophages (Fig. 2D).

Macrophage chemoattractant chemokines MCP-1 (CCL2) and RANTES (CCL5): In a rat model of cancer and in human monocytes *in vitro,* Josephs and colleagues demonstrated, that IgE crosslinking triggered a TNFα/MCP-1 axis, which resulted in recruitment of macrophages into tumours [9]. Here, crosslinking of cell-bound IgE produced no modulation of MCP-1 supernatant concentration in either M0 or M1 macrophages. However, M2 macrophages, responded to IgE activation by enhancing the release of MCP-1. M0 and M2 but not M1 macrophages increased production of RANTES with IgE crosslinking, with the peak concentrations following IgE stimulation corresponding approximately to the baseline concentration in M1 cells (Fig. 2E).

Together these data demonstrate that crosslinking of cell-bound IgE triggered enhanced expression of cytokines TNFα, IFNγ, IL-12, IL-1β, IL-6, IL-10, IL-13 and of the chemokines MIG (CXCL9), I-TAC (CXCL11) and RANTES (CCL5) by M2 and M0 macrophages. IgE crosslinking on M2 macrophages but not on M0 or M1 subsets, triggered enhanced secretion of the chemoattractant MCP-1 (CCL2). On the other hand, M1 macrophages appeared to retain their cytokine/chemokine expression upon IgE activation including expression of pro-inflammatory mediators such as IFNγ and IL-12 (Table 1).

**Table 1:**
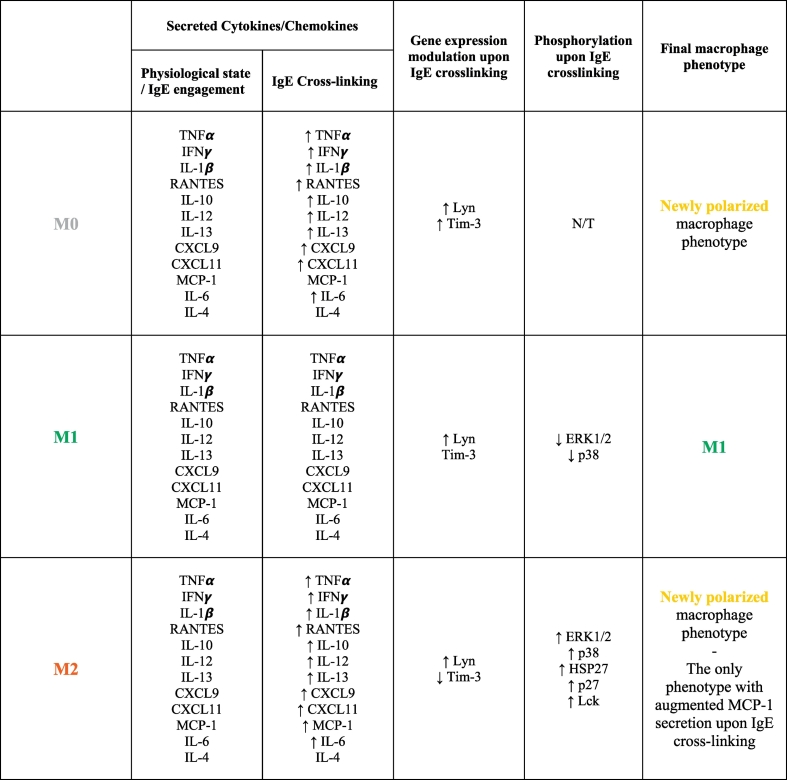
Summary of IgE antibody-mediated effects on human macrophage phenotypes

List of soluble mediators released, gene expression and kinase phosphorylation modifications engendered on human macrophage subsets upon IgE crosslinking. Quiescent M0 and M2 macrophages cross-linked with IgE antibodies acquire typical M1 marker features and are skewed towards a new polarisation status (yellow macrophage). IgE cross-linking on M1 macrophages retains a pro-inflammatory, anti-tumour polarisation status. The five most perturbed kinases amongst 43 analysed were reported in this table. The red arrow indicates increase/decrease of release, expression or phosphorylation level compared to baseline conditions.

### 3.3 Crosslinking of IgE on M1 and M2 macrophages regulates phosphorylation levels of protein kinases downstream of FcεRI signalling pathway

**Fig. 3:**
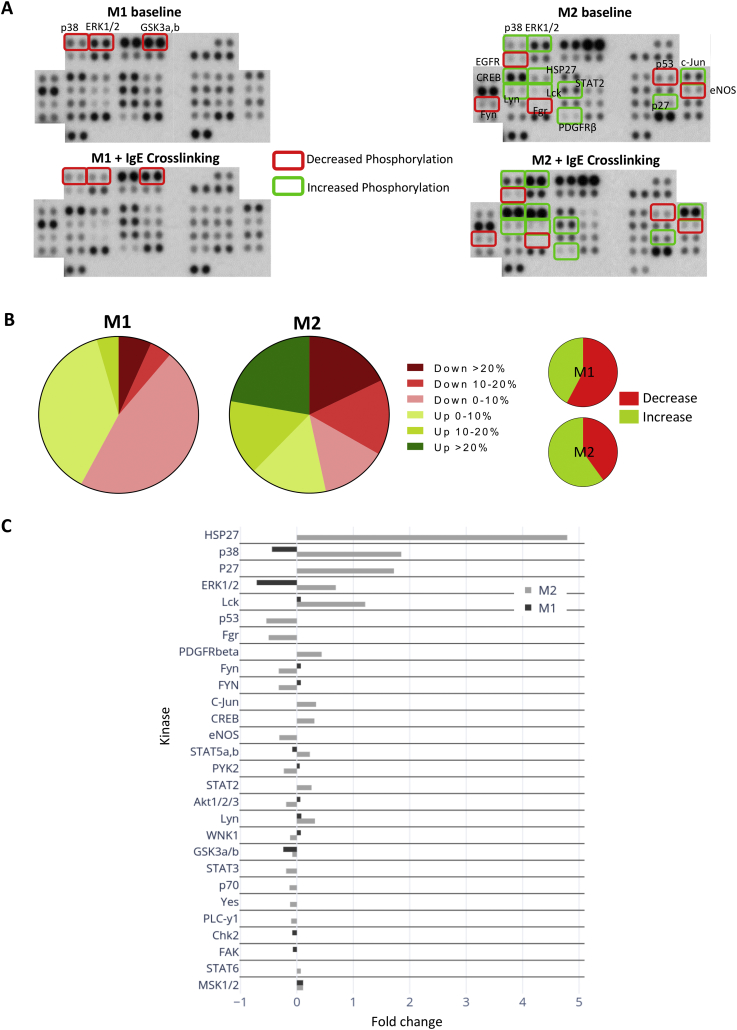
Kinase phosphorylation is differentially altered between M1 and M2 macrophages upon IgE cross-linking. (a) Images from Proteome Profiler Human Phospho-Kinase Array (decrease in phosphorylation marked in red; increase in phosphorylation marked in green). Each kinase is spotted in duplicate. (b) Schematic representation of the percentage of increase and decrease in phosphorylation levels in M1 and M2 macrophages upon IgE cross-linking. (c) Pixel densitometry analysis was expressed as fold change comparing the sample subjected to IgE cross-linking to the corresponding non-treated sample. (For interpretation of the references to colour in this figure legend, the reader is referred to the web version of this article.)

Crosslinking of differentiated macrophages M1 and M2 with IgE/anti-IgE resulted in modification of the phosphorylation levels of intracellular kinases, analysed *via* a phosphorylation array (Fig. 3A). Amongst 43 kinases analysed, overall 29 were significantly perturbed in IgE-stimulated macrophages. M2 macrophages were characterised by a marked increase in phosphorylation levels compared to the M1 subset, which in contrast displayed a decrease of phosphorylation for the majority of kinases analysed (Fig. 3B). M2 macrophages displayed a marked increase (1.25–5-fold changes) in the phosphorylation of HSP27, p38, p27, ERK1/2 and Lck kinases following crosslinking. Crosslinking of IgE on M1 macrophages induced downregulation of p38, ERK1/2, STAT5a/b, GSK3a/b, Chk2 and FAK phosphorylation, while some kinases showed upregulated phosphorylation of maximum 0.2-fold change compared to baseline (Fig. 3C). MSK1/2 was the only kinase displaying slightly increased phosphorylation of 0.2-fold change in both macrophage subsets (Fig. 3C).

**Fig. 4:**
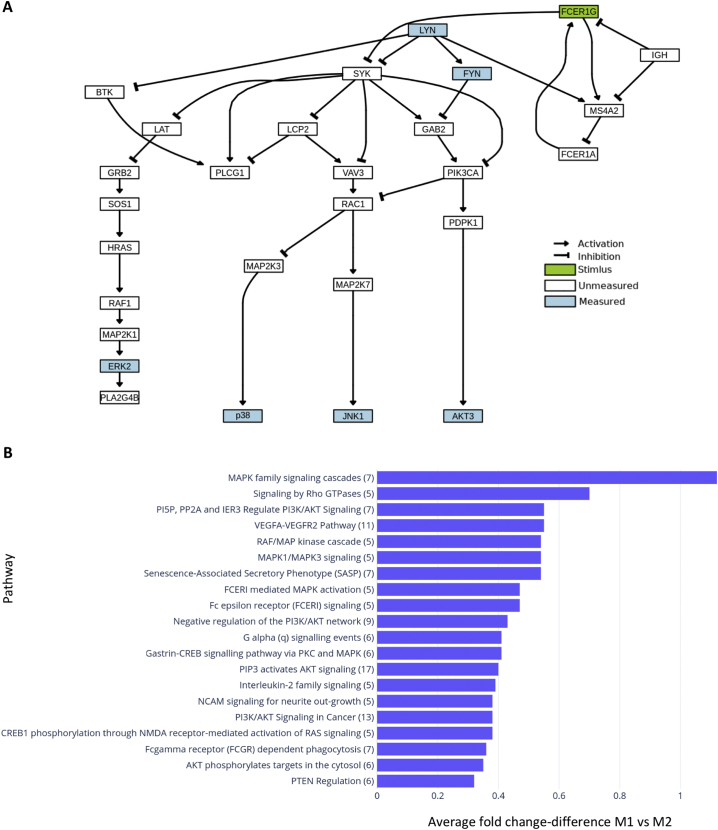
Human M1 and M2 macrophages display differential signalling cascade upon IgE engagement. (a) FcERI pathway network generated *via* KEGG database. Highlighted in blue are kinases analysed *via* Human Phospho-Kinase Antibody Array. (b) List of implicated pathways upon IgE cross-linking, represented as the average fold change of implication between M1 and M2 macrophages. Data generated *via* Reactome. (For interpretation of the references to colour in this figure legend, the reader is referred to the web version of this article.)

In order to investigate whether IgE stimulation influenced FcεRI signalling, commonly known to be activated on mast cells, we interrogated the publicly-available KEGG database to depict the FcεRI signalling pathway. Molecular species whose phosphorylation level was measured *ex vivo* from IgE-stimulated differentiated human macrophages are depicted in blue (Fig. 4A). Since the FcεRI signalling pathway in human macrophages is relatively unexplored, we interrogated a publicly available database (Reactome) with the proteome phospho-profiler dataset to dissect differences between M1 and M2 macrophage signalling cascades upon IgE crosslinking. IgE crosslinking on macrophages triggered phosphorylation of kinases known in mast cells to be found downstream of the FcεRI pathway network (generated *via* the KEGG database) (Fig. 4A). Furthermore, a number of signalling pathways appeared to be differentially activated following IgE crosslinking in the two macrophage subsets (VEGFA-VEGFR2 pathway, PI3K/AKT and Rho GTPases signalling amongst others), with the MAPK family pathway being the most differentiating between classically- and alternatively-activated macrophages (Fig. 4B). These findings confirm for the first time that IgE stimulation potentiates activation of kinases downstream of the FcεRI signalling pathway in macrophages and reflect differential intracellular mechanisms specific for each alternatively-activated and classically-macrophage subset triggered by activation of FcεRI.

### 3.4 IgE crosslinking on macrophages modulates gene expression of *Lyn* and *Tim-3* and *Lyn* gene expression is associated with a favourable prognosis in solid tumours

**Fig. 5:**
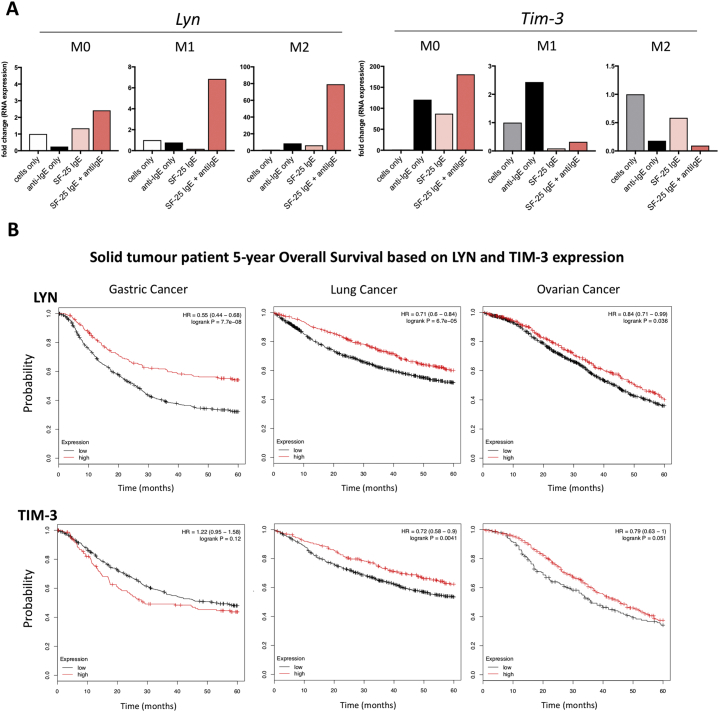
Effects of IgE cross-linking on macrophage gene expression and correlation of LYN and TIM-3 expression of tumour patient prognosis. (a) Differentiated M0, M1 and M2 macrophages were treated with: SF-25 IgE alone, anti-human IgE or both. The effects of IgE cross-linking on gene expression of LYN and TIM-3 were detected by performing RT-PCR on mRNA content of treated and lysed cells. Values are expressed as fold change compared to the untreated cell sample of one single preliminary experiment. (b) Association between mRNA expression of crucial elements of Fc*ε*RI pathway LYN and TIM-3 and 5-year survival of patients in cohorts of gastric, lung and ovarian cancer were analysed. Graphs show Kaplan-Meyer survival curves upon discrimination between high and low expression levels of genes of interest *Lyn* and *Tim-3*. Threshold was set to the upper quartile of patients' gene expression data: “high” expression represents the upper quartile, “low” expression encompasses the remaining data of the set.

We next investigated the gene expression of the protein kinase *Lyn* and the checkpoint molecule *Tim-3* (T cell or transmembrane immunoglobulin and mucin domain protein 3), both known to participate in the FcεRI signalling pathway in mast cells [31]. Upon IgE crosslinking, *Lyn* mRNA expression increased to 2-, 7- and 70-fold in M0, M1 and M2 macrophages, respectively (Fig. 5A, left). M2 macrophages downregulated *Tim-3* expression upon IgE activation, while M0 macrophages increased *Tim-3* gene expression by 150-fold in response to IgE crosslinking compared to untreated cells (Fig. 5A, right). Together these data demonstrate that the FcεRI pathway can be activated with IgE on macrophage subsets *ex vivo* and gene expression of *Lyn* and *Tim-3* are differentially regulated by IgE on distinct macrophage subsets.

To gain insights into a potential clinical relevance of IgE/FcεR signalling through *Lyn* and *Tim-3,* we interrogated publicly-available gene expression datasets for solid tumours of different origins (Fig. 5B). We found significant association of improved 5-year overall survival with elevated expression of *Lyn* in gastric (*P* = 7.7e-08), lung (*P* = 6.7e-05) and ovarian (*P* = .036) cancers. These findings suggest that enhanced *Lyn* gene expression - one of the downstream signalling effects of IgE crosslinking its FcεR on alternatively-activated and classically-activated macrophages - may be associated with better prognosis in cancer patients. On the other hand, the association of *Tim-3* gene expression with clinical outcomes appeared dependent on the tumour type: specifically, higher levels of *Tim-3* expression in lung and ovarian cancers correlated with improved patient prognosis, while it was associated with less favourable overall survival in patients with gastric cancer. This suggests that Tim-3 may exert different functions in different malignancies. These findings may be significant in future IgE class-mediated immune cell activation in different solid tumour types.

### 3.5 M0, M1 and M2 derived human macrophages can be activated by anti-tumour IgE to kill cancer cells

**Fig. 6:**
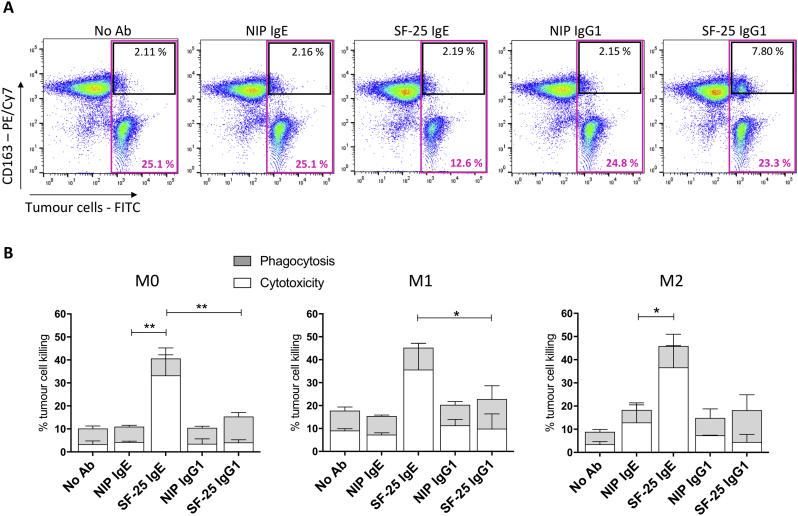
IgE antibodies activate *ex vivo* monocyte-derived macrophages towards A-2058 melanoma cells. The tumour cell killing assay was performed in order to investigate a) the ability of IgE antibodies to activate *ex vivo* derived macrophages and b) whether the different phenotypes could reflect into different anti-tumour activity. (a) Representative flow cytometry plots depicting the gating strategy applied for detection of cytotoxic and phagocytic activity of macrophages. The black gate highlights the double positive cells, indicating phagocytic activity (CFSE+ tumour cells internalized by macrophages). The purple gate emphasizes the loss in the total number of tumour cells when incubated with SF-25 IgE antibody. (b) Tumour cell killing was investigated in PBS control conditions, with 5 μg/ml of SF-25 I.E. SF-25 IgG1 and negative (non-specific) (NIP IgE or IgG1) antibody controls. Values are represented as percentage of tumour killing (average of 2 independent experiments ± SEM; each condition was tested in triplicate). Statistical analysis obtained performing One-way ANOVA coupled with Bonferroni's post-test; ** p-value = .0012, * p-value = .01 (M1) and 0.03 (M2) (* p-value = .01) * = p-value<.05, ** = p-value<.01. (For interpretation of the references to colour in this figure legend, the reader is referred to the web version of this article.)

We assessed whether human macrophages derived *ex vivo* can be activated by IgE to kill cancer cells by applying a three-colour flow cytometric assay to detect cytotoxicity and phagocytosis (14) (Fig. 6A). The anti-tumour antibody SF-25 IgE was successful in engaging all three macrophage subsets, M0, M1 and M2 to mediate tumour antigen-specific ADCC of A-2058 cancer cells compared with non-specific antibody-treated or no antibody control cells (Fig. 6B). SF-25 IgE triggered approximately 30% of tumour cell-killing above controls. On the other hand, treatment of macrophages and A-2058 tumour cells with SF-25 IgG1 isotype antibody did not produce effective tumour cell killing above isotype controls. These findings support the notion that IgE antibodies can activate human macrophages to kill tumour cells *ex vivo*, but also that both M1 and M2 subsets can engage with antibodies of the ε class and target cancer cells by exerting antigen-specific cytotoxic activity.

**Fig. 7:**
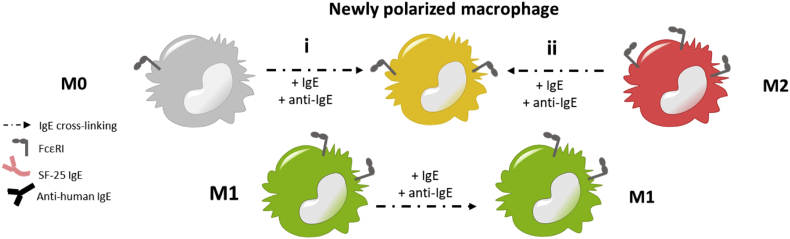
IgE antibody-mediated re-programing of human macrophages. Schematic picture illustrating the suggested model of repolarization of human macrophages *via* engagement and activation by IgE antibodies: quiescent M0 macrophages and anti-inflammatory M2 macrophages are skewed towards a new polarisation phenotype, displaying both pro- and anti-inflammatory features.

In summary, crosslinking IgE antibodies on the surface of human macrophages influenced their polarisation status (Fig. 7), leading quiescent macrophages to acquire M1-like features, M1 macrophages to retain their pro-inflammatory status and skewing differentiated M2 macrophages towards a pro-inflammatory phenotype, with measurable anti-tumour activity when tumour antigen-specific IgE could target macrophages against cancer cells.

## 4 Discussion

Macrophages represent a major component of the tumour microenvironment in many solid cancers. Their phenotypes and activation states can be influenced in the TME in response to environmental stimuli, so that different macrophage subsets could orchestrate different and often opposite aspects of the immune response, striking a balance between anti-tumour immunity and wound healing or inflammation. The potential of antibodies to influence the dynamics and polarisation states of these cells against cancer may offer opportunities to tip the balance in favour of host immunity. However, the mechanisms of human macrophage-associated immune complex formation engendered by different antibody isotypes, and especially through the IgE/FcεR (IgE FcR) axis, remain insufficiently understood.

We have previously reported that systemic treatment with anti-tumour IgE reduced tumour growth in different *in vivo* models of cancer, associated with significant recruitment of macrophages towards tumours [9,29]. However, the mechanisms by which therapeutic antibodies of the IgE class can engage, influence and activate tissue macrophage populations, especially those likely to form part of the tumour microenvironment, remain unexplored. In this study we investigated whether IgE stimulation may influence the polarisation and activation states of classically- and alternatively-activated macrophage populations. For this purpose, we derived human macrophages displaying the typical markers of M1 (classically-activated) or M2 (alternatively-activated) phenotypes and cytokine profiles [32]. We therefore explored the presence of cognate Fcε receptors on the surface of classically- and alternatively-activated macrophages and interrogated the effect of IgE engagement and of crosslinking of macrophage-bound IgE on each subset.

Around 40% of M2 macrophages expressed the high affinity IgE receptor FcεRI, while approximately 20% of cells in each subset expressed the low affinity IgE receptor CD23. Macrophage cell surface-bound IgE crosslinked on alternatively-activated macrophages triggered increased expression of the co-stimulatory molecule CD80, a typical M1 macrophage marker. Upregulation of the co-stimulatory molecule CD80 on the surface of professional antigen-presenting cells (APC) including macrophages, may signify cell maturation and also confer capacity for effective antigen presentation and may point to the potential of IgE class antibodies to promote macrophage maturation, polarisation and APC functions. These findings are consistent with previously described evidence of: a) infiltration of immune cells, including macrophages, in human ovarian cancer patient-derived xenograft residual tumours in immunocompromised mice treated with an anti-tumour IgE and human PBMCs as effector cells; and b) higher recruitment of monocyte/macrophages into tumours and enhanced expression of CD80+ rat macrophages into syngeneic tumours in an immunocompetent rat model following systemic treatment with rat MOv18 IgE [4,9]. Furthermore, in our study, IgE stimulation did not interfere with the expression of CD163 on either macrophage population. In concordance, we previously reported that in rats treated with anti-tumour IgE, tumour-infiltrating rat macrophages did not upregulate the M2 marker CD163 [9]. Together, these indicate that activation of the IgE/FcεR pathway may influence macrophage phenotype and maturation.

We furthermore explored whether IgE stimulation may promote differential activation of specific macrophage subsets. In M1 macrophages, IgE crosslinking retained the production of key pro-inflammatory cytokines including IFNγ and IL-12. In concordance, in Josephs et al., we recently demonstrated upregulation of pro-inflammatory immune-associated pathways including IL-12 and NK-cell immune activation signatures in the lungs of tumour-bearing rats treated with MOv18 IgE [10]. Considering these findings, it is possible that IgE engagement and crosslinking may at least retain M1 macrophages in a manner consistent with their known pro-inflammatory and antigen-presenting functions.

Importantly, secretion of IL-1β, IL-6, IL-10, IL-12, IL-13, IFNγ, RANTES, MIG and I-TAC was triggered by crosslinking of cell-bound IgE on quiescent M0 and on M2 macrophages. This may suggest that the two subsets might feature similar molecular mechanisms in their ability to regulate activation of the FcεRI pathway. Furthermore, production of all soluble mediators tested was either retained at the same level or increased with IgE-crosslinking. Although the cytokine panel secreted by M0 and M2 after crosslinking with IgE did not correspond to a defined macrophage subtype, the mediator signature displayed by IgE-stimulated cells may represent a newly polarized macrophage subset secreting both pro- and anti-inflammatory mediators, as well as chemoattractant factors (Table 1). An additional result that also points at the development of a macrophage state with activatory features is increased release of CXCL9 with IgE stimulation in M0 and M2 macrophages: CXCL9 represents an important anti-angiogenic factor, known to be predominantly expressed by the M1 phenotype [33]. Since CXCL9 is a direct counter-regulatory mediator of VEGF signalling known to influence macrophage polarisation [34], future studies may also directly assess the impact of IgE antibody stimulation on the expression of VEGF by different macrophage subsets.

We also report that IgE crosslinking on both M0 and M2 macrophages triggered enhanced levels of the pro-inflammatory M1 cytokine TNFα, which this had different effects in M0 and M2 macrophages in the relation to the production of the macrophage chemoattractant MCP-1. M2 macrophages, upon IgE treatment, enhanced the production and the release of MCP-1. In line with the findings presented in this work, upregulation and the potential roles of TNFα and MCP-1 have been described by Josephs and colleagues. Our previous findings with the anti-tumour IgE antibody MOv18 in a rat tumour model *in vivo* suggested that tumour antigen-specific IgE treatment influences the spatial distribution and activation of macrophages around tumour lesions. Systemic IgE treatment in this model was also associated with a specific cytokine signature, featuring elevated levels of TNFα, MCP-1 and IL-10 in the tumour microenvironment and upregulated expression of TNFα by tumour-associated macrophages in rat lungs. Furthermore, *ex vivo* crosslinking of MOv18 I.E. triggered TNFα upregulation by human monocytes. TNFα stimulation triggered production of MCP-1 by macrophages and also by tumour cells of different origins. MCP-1 triggered monocyte and macrophage infiltration in the tumour microenvironment. These studies demonstrated the significance of the TNFα/MCP-1 axis in the modulation of cellular populations and functions in the TME [9,35]. Our findings that M0 and M2 macrophages can be stimulated to secrete TNFα and that alternatively activated M2 macrophages are the only cell type that upregulated MCP-1 upon crosslinking by IgE, suggest that IgE has the capacity to specifically stimulate this normally anti-inflammatory subset to a more mature, further activated phenotype, which bears some characteristics normally associated with the classically-activated M1 macrophages. Further investigation of the effects of IgE antibodies on the expression of additional soluble mediators such as IL-17A, and other family members, and IL-37, reported to influence macrophage phenotype, may shed light into the impact of IgE-FcεRs interactions in the tumour microenvironment [36,37].

Analysis of gene expression of the protein kinase *Lyn* and *Tim-3* both part of the FcεRI signalling pathway and known to regulate effector cell activation [38], showed that crosslinking of IgE on macrophages upregulated *Lyn* gene expression in M0, M1 and M2 macrophage subtypes. This may be significant in the cancer context since *in vivo* studies demonstrated that a loss-of-function of *Lyn* may predispose to tumorigenesis [39], and transgenic mouse models lacking *Lyn* were characterised by defective macrophage populations [40]. The retention and upregulation of *Lyn* expression on all macrophage subsets may therefore signify the importance of activating macrophages by antibodies of the IgE isotype, which could potentially confer protection from tumour growth. The potential of activating the FcεR downstream pathway, and by inference anti-tumour IgE engagement of immune effector cells may have clinical significance, since higher intra-tumoural *Lyn* expression in gastric, lung and ovarian cancer is associated with more favourable prognosis. On the other hand, engagement and crosslinking of IgE induced a reduction in *Tim-3* gene expression in M2 macrophages, while *Tim-3* was upregulated in M0 macrophages. Tim-3, known to participate in multiple suppressor pathways of anti-tumour responses, can be expressed by cancer cells and may support alternatively-activated macrophage polarisation. Simultaneous blocking of Tim-3 and PD-1 in infectious diseases was shown to restore lymphocyte functions and it was recently hypothesised that blocking Tim-3 may support the anti-tumour functions by macrophages [41]. IgE crosslinking and subsequent reduction in *Tim-3* gene expression in M2 macrophages may induce immune cell activation, in line with what was observed with T lymphocytes upon treatment with anti-PD-1 and anti-Tim-3 antibodies. Therefore, reduction of Tim-3 expression on M2 macrophages upon IgE treatment may be further explored in the context of cancer immunotherapy and macrophage functions in the TME.

In summary, analysing surface marker expression, soluble mediator release, gene expression and protein phosphorylation on human macrophages derived *ex vivo* upon IgE crosslinking may provide a new insight on this antibody class that may have relevance in tissues and perhaps in TME. Quiescent M0 macrophages, when crosslinked with IgE, featured: upregulation of the classic M1 surface marker CD80, increased production of pro-inflammatory cytokines and chemoattractant mediators, increased expression of the kinase *Lyn* and other downstream signalling molecules including Erk1/2 and p38. M1 macrophages retained their pro-inflammatory status upon activation *via* IgE antibodies, which led to increased expression of *Lyn* downstream of the FcεRI pathway. The crosslinking of IgE antibodies on M2 macrophages was characterised by a significant increase in the production of the chemoattractant, pro-inflammatory molecule MCP-1 and the enhanced phosphorylation and gene expression of *Lyn*. Therefore, crosslinking of IgE on human macrophages confers M0 and M2 subsets features more likely associated with classically-activated macrophages (Fig. 7), leading to polarized macrophage phenotypes. Our functional assays indicate that anti-tumour IgE antibodies can engage human macrophages to elicit an effector function response against cancer cells. We show for the first time that IgE can activate macrophages of different polarisation states to kill cancer cells. This supports the notion that tissue type macrophages, including those subsets commonly associated with tumour lesions, could be engaged and re-educated by anti-tumour IgE antibodies, and importantly, that IgE could unleash their cytotoxic potential against target tumour cells.

The anti-cancer potential of IgE class antibodies has been reported in multiple studies in the emerging field of AllergoOncology, concerned with the links between Th2 and IgE responses with cancer [42,43]. Reports include an IgE antibody recognising the cell surface tumour antigen HER2/*neu* which restricted tumour growth in human FcεRI transgenic mice. Furthermore, Fazekas-Singer et al. reported the development of a canine IgE targeting the epidermal growth factor receptor (EGFR) shown to engage and activate canine macrophages more effectively than the equivalent IgG1 to target and kill tumour cells *in vitro* [44]*.* Human adipose tissue-derived mast cells (ADMC) can be specifically activated to display tumoricidal activity against breast cancer cells when engaged with tumour antigen-specific IgEs [45]. Our own studies have demonstrated the capacity of an anti-FRα IgE antibody (MOv18) to restrict tumour growth in several model systems and to recruit monocytes and macrophages against tumours, supporting the progress of this agent to a first-in-class Phase I clinical trial in oncology (NCT02546921, www.clinicaltrials.gov).

Taken together, our findings point to the potential of anti-tumour IgE antibodies not only to mediate immune effector cell-mediated killing of cancer cells, but also to impact on immune stroma, influencing macrophage phenotype and maturation states. Our findings provide new insights into the interactions between human macrophages and IgE class antibodies. This may be highly significant in the clinical development of new immunotherapy strategies targeting these often tissue- and tumour-resident cells and may point to previously-unappreciated cascades with potential to activate and re-educate immune stroma.

**Supplementary Fig. 3:**
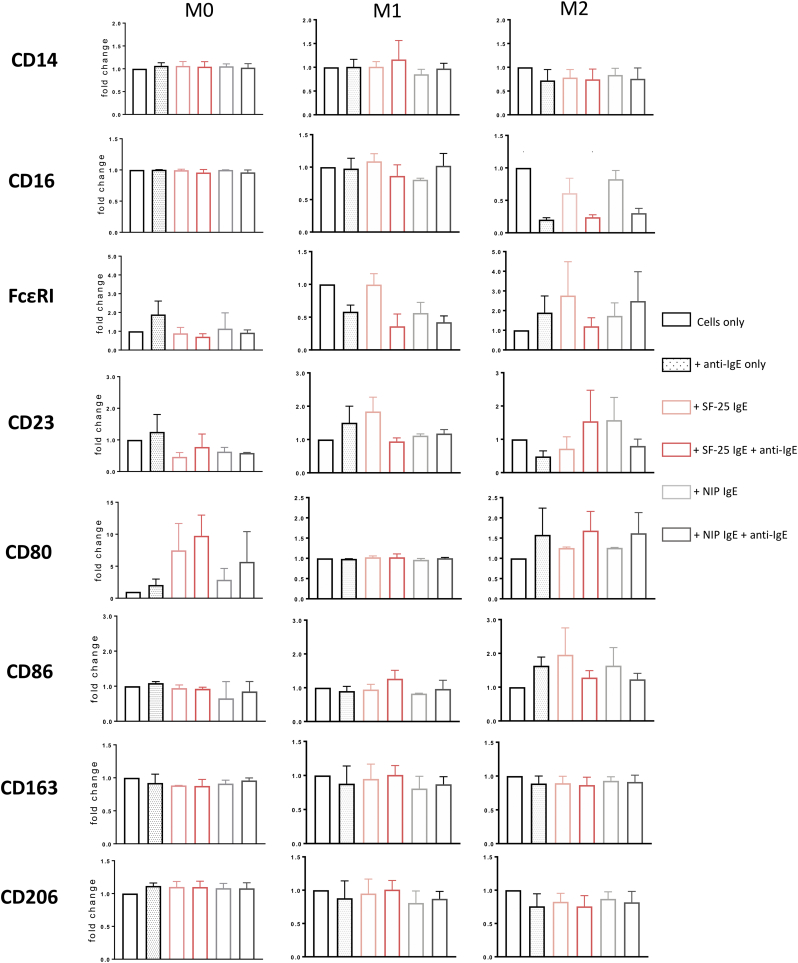
Cross-linking of cell-bound IgE regulates surface marker levels of macrophages Differentiated macrophages (n = 3) were incubated with SF-25 IgE or NIP IgE antibody alone or in combination with anti-human IgE antibody. Monocyte, M1 and M2 typical surface marker levels were analysed before and after treatment by staining the cells with fluorescently labelled antibodies targeting CD14, CD16, Fc*ε*RI, Fc*ε*RII (CD23), CD80, CD86, CD163 and CD206. Values are expressed as fold change compared to the non-treated sample ± SEM, based on a number of 3 independent experiments.
